# COVID-19-associated autoimmune encephalitis: A case report and literature review

**DOI:** 10.1097/MD.0000000000039533

**Published:** 2024-09-20

**Authors:** Yang-Chuan Chen, Shu-Ting Hong, Yuan-Feng Chen, Pan Lin, Xiao-Xin Chen, Yang-Zong Wu

**Affiliations:** a Department of Emergency, The Second Hospital of Longyan, Longyan, Fujian Province, China; b Department of Neurosurgery, National Regional Medical Center, Binhai Campus of the First Affiliated Hospital, Fujian Medical University, Fuzhou, Fujian Province, China; c Department of Neurosurgery, The Second Hospital of Longyan, Longyan, Fujian Province, China; d Department of Neurology, The Second Hospital of Longyan, Longyan, Fujian Province, China.

**Keywords:** autoimmune encephalitis, infection, novel coronavirus disease 2019, steroid pulse therapy

## Abstract

**Rationale:**

This article reports a case of coronavirus disease (COVID-19)-associated autoimmune encephalitis (AE) and reviews the relevant literature to investigate the clinical manifestations, auxiliary inspection, diagnosis and treatment, and prognosis of AE associated with COVID-19.

**Patient concerns:**

A 68-year-old female with fatigue developed altered consciousness after 2 days of fever, thereafter testing positive for COVID-19. The protein levels in the lumbar puncture cerebrospinal fluid were elevated, and cranial magnetic resonance imaging (MRI) scan indicated T2-weighted hyperintensity in the temporal lobe.

**Diagnoses:**

The patient was diagnosed with COVID-19-associated AE.

**Interventions:**

After admission, the patient received pulse steroid therapy with methylprednisolone. Additionally, gastric protection, blood glucose control, nutritional support, and other treatments were administered.

**Outcomes:**

The **s**ymptoms were significantly relieved by steroid pulse therapy. At the 3-month follow-up, the patient had recovered completely without any obvious discomfort.

**Lessons:**

The possibility of AE should be considered if neurological symptoms occur a few days after infection with COVID-19, with early diagnosis and immediate steroid pulse therapy resulting in better outcomes.

## 1. Introduction

The end of 2019 marked the emergence of a global pandemic of coronavirus disease (COVID-19), which has given rise to not only respiratory symptoms but also nervous system-associated complications in infected individuals. COVID-19-associated viral encephalitis and autoimmune encephalitis (AE) are common neurological complications.^[1]^ However, reports of COVID-19-associated AE remain scarce in the literature. In this study, we reported a case of COVID-19-associated AE and conducted a literature review to investigate the clinical manifestations, auxiliary inspection, diagnosis, treatment, and prognosis of AE associated with COVID-19 infection. This study was approved by the Medical Science Research Ethics Committee of the Second Hospital of Longyan, Fujian Province.

## 2. Case presentation

### 2.1. Clinical manifestation

A 68-year-old female was admitted to the hospital on January 6, 2023, with symptoms of fatigue for 4 days and hampered speech and walking instability for 2 days. Fatigue and fever occurred 4 days before admission with no obvious inducing factors, with the temperature reaching about 38°C without cough, expectoration, or other discomfort. A diagnosis of “cold” was considered at a local clinic, and the symptoms relieved after intravenous infusion therapy. However, 2 days later, the patient presented with the loss of a capacity for recognizable speech, an inability to walk, a decline in consciousness, cough, and occasional expectoration. No relief was observed following treatment with cefotaxime sodium at a local clinic. The patient had a history of diabetes and received oral medications, including metformin sustained-release tablets. Physical examination indicated a temperature of 36.6°C, a pulse of 80 times/min, a respiratory rate of 21 times/min, and a blood pressure of 141/80 mm Hg. Her consciousness was classified as mental confusion, with a Glasgow Coma Scale score of 12 points (eye response 3, verbal response 4, motor response, 5). The bilateral pupils had a diameter of 3 mm and were sensitive to light reflection. Neck resistance and hypertonia were detected, and the patient was unable to cooperate during the examination of muscle strength. Kernig sign and bilateral pathological reflexes were also detected.

### 2.2. Auxiliary examination and diagnosis

**Figure 1.**
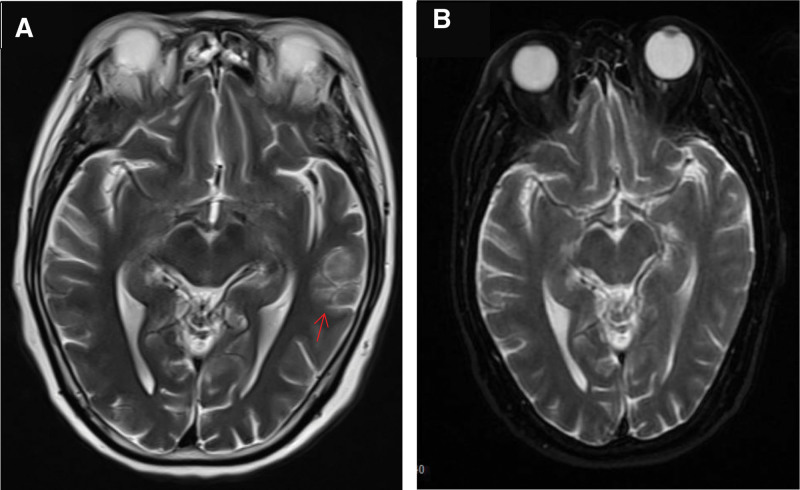
(A) T2-weighted MRI indicated a lesion with hyperintensity signal in left temporal lobe (red arrow). (B) T2-weighted MRI indicated the lesion in left temporal lobe disappeared after 10 d of treatment.

A novel coronavirus nucleic acid test for COVID-19 yielded positive results. Computed tomography scan showed no abnormality in the brain, and there were minor inflammatory manifestations in the apicoposterior segment of the left upper lobe and the subpleural area of the right lower lobe. A cranial magnetic resonance imaging (MRI) T2-wighted scan indicated hyperintensity in the area of the sulci in the left temporal lobe, and no other abnormalities were observed [Fig. 1A]. The patient’s leukocyte, C-reactive protein, and procalcitonin levels in the blood were normal. Cerebrospinal fluid (CSF) examination through lumbar puncture showed normal pressure and color, with a leukocyte count of 20/L, total protein levels of 95.33 mg/dl, normal glucose and chloride levels, and negative autoimmune antibody test results.

The patient was diagnosed with COVID-19-associated AE.

### 2.3. Treatment and outcomes

After admission, the patient received steroid pulse therapy with intravenous methylprednisolone prescribed intravenously (1.0 g/d) for 3 days. Three days later, the steroid reduced to 60 mg/day, and reduced at a rate of half reduction every week until discontinuation 1 month later. Additionally, gastric protection, blood glucose control, nutritional support, and other treatments were administered. After 1 dose of methylprednisolone, the patient regained consciousness, was able to eat by themselves, and hypertonia was significantly relieved. CSF examination was normal 1 week later, and the T2-hyperinsensity signal in the left temporal lobe on MRI disappeared after 10 days [Fig. 1B]. At the 3-month follow-up, the patient had recovered completely without any obvious discomfort.

## 3. Discussion

At present, reports of COVID-19-associated AE in the literature are scarce, with the majority being case reports. Despite this, some researchers have been able to summarize this condition based on the existing literature, classifying it into limbic encephalitis, anti-N-methyl-d-aspartate (NMDA) receptor encephalitis, new-onset refractory status epilepticus-associated encephalitis, steroid-responsive encephalitis, and unknown types of encephalitis.^[1]^ COVID-19-associated AE usually occurs a few days after COVID-19 infection, with neuropsychiatric symptoms including altered consciousness, mental confusion, speech disorders, cognitive disorders, and epilepsy. Some studies have reported that acute COVID-19 induces neurological and neuropsychiatric manifestations in pediatric patients.^[2,3]^ Physical examination has revealed neck resistance and limb hypertonia. On CSF examination, the White blood cell count usually increases slightly, and the protein level increases with a positive oligoclonal band. CSF autoimmune antibodies in the CSF. MRI scans have been used to detect cerebral T2-hyperintensity signal, while epileptiform discharges have been detected on the electroencephalograms of some patients.^[1,4–7]^ However, patients often have no obvious symptoms of fever or a significant increase in inflammatory indicators. At present, the primary treatment is steroid and gamma globulin pulse treatment,^[6,7]^ with early detection and immediate treatment having been shown to achieve better outcomes.^[1]^

This case involved an elderly female patient who developed fatigue and fever 4 days before admission with a positive COVID-19 nucleic acid test result during admission. The patient was considered to have been infected COVID-19 at 4 days before admission. Two days after symptom onset, the patient presented with altered consciousness, neck resistance, and hypertonia in the limbs, and tests indicated increased White blood cell and protein levels in CSF. The patient had no fever and normal inflammatory indicators, which ruled out viral encephalitis.^[4]^ Although the CSF and serum tested negative for AE-related antibodies, we still considered COVID-19-associated AE^[1]^ instead of COVID-19-associated viral encephalitis according to previous reports on the pathogenesis of AE. After 1 day of steroid pulse therapy, the symptoms were significantly relieved. Examination on the seventh day showed normal CSF, and the T2-hyperintensity signal lesion in the left temporal lobe disappeared at the tenth day after treatment. Thus, based on the course of the disease and a review of the relevant literature, this patient was diagnosed with steroid-sensitive COVID-19-associated AE.^[1,6]^

This case represents the successful treatment of COVID-19-associated AE. At present, COVID-19 has yet to be eliminated and may continue to spread globally. Although cases of COVID-19-associated AE are rare, they should not be overlooked. AE should be considered when patients show neurological symptoms a few days after COVID-19 infection, which are characterized by positive meningeal irritation signs, hypertonia, and normal peripheral blood inflammatory indicators. The diagnosis can be further confirmed by CSF examination, MRI, and electroencephalography.

## Author contributions

**Conceptualization:** Yang-Zong Wu.

**Data curation:** Yuan-Feng Chen, Pan Lin.

**Funding acquisition:** Yang-Zong Wu.

**Writing – original draft:** Yang-Chuan Chen, Xiao-Xin Chen.

**Writing – review & editing:** Yang-Chuan Chen, Shu-Ting Hong.
